# The availability of water associated with glycogen during dehydration: a reservoir or raindrop?

**DOI:** 10.1007/s00421-017-3768-9

**Published:** 2017-12-01

**Authors:** Roderick F. G. J. King, Ben Jones, John P. O’Hara

**Affiliations:** Institute for Sport, Physical Activity and Leisure, Headingley Campus, Leeds Beckett University, Leeds, West Yorkshire LS6 3QS UK

**Keywords:** Hydration, Osmolality, Fluid, Nutrition

## Abstract

**Purpose:**

This study evaluated whether glycogen-associated water is a protected entity not subject to normal osmotic homeostasis. An investigation into practical and theoretical aspects of the functionality of this water as a determinant of osmolality, dehydration, and glycogen concentration was undertaken.

**Methods:**

In vitro experiments were conducted to determine the intrinsic osmolality of glycogen–potassium phosphate mixtures as would be found intra-cellularly at glycogen concentrations of 2% for muscle and 5 and 10% for liver. Protected water would not be available to ionic and osmotic considerations, whereas free water would obey normal osmotic constraints. In addition, the impact of 2 L of sweat loss in situations of muscle glycogen repletion and depletion was computed to establish whether water associated with glycogen is of practical benefit (e.g., to increase “available total body water”).

**Results:**

The osmolality of glycogen–potassium phosphate mixtures is predictable at 2% glycogen concentration (predicted 267, measured 265.0 ± 4.7 mOsmol kg^−1^) indicating that glycogen-associated water is completely available to all ions and is likely part of the greater osmotic system of the body. At higher glycogen concentrations (5 and 10%), there was a small amount of glycogen water (~ 10–20%) that could be considered protected. However, the majority of the glycogen-associated water behaved to normal osmotic considerations. The theoretical exercise of selective dehydration (2 L) indicated a marginal advantage to components of total body water such as plasma volume (1.57% or 55 mL) when starting exercise glycogen replete.

**Conclusion:**

Glycogen-associated water does not appear to be a separate reservoir and is not able to uniquely replete water loss during dehydration.

## Introduction

The precise measurement of fluid balance to monitor the hydration status of an individual has received substantial interest over the last century (Cheuvront and Kenefick 2014). Life events cause fluid imbalance, for example, during physical activity, sweat loss typically exceeds fluid intake (Jones et al. 2015, 2016a, b). Furthermore, individuals lose water during respiratory exchange and in the production of urine. Acutely, the necessity to drink (in excess of fluid losses) may lead to hyperhydration (Bargh et al. 2016). The balance of mechanisms acting on regulated variables as well as controlled variables operating to an apparent set point of osmolality usually affects a mild oscillation around a specific body water content (Tam and Noakes 2013).

Fluid imbalances inevitably affect total body water (TBW), specifically intracellular fluid (ICF) and extracellular fluid (ECF) compartments. The source of the fluid loss is typically determined by osmotic disturbance (Baker and Jeukendrup 2014). There are well-evolved mechanisms to modulate the effects of sweat loss and consequent impact on ECF. Hypotonic losses (i.e., sweat, Buono et al. 2008) increase ECF osmolality, and consequently, fluid moves from ICF to equilibrate the change, known as intracellular dehydration (Cheuvront and Kenefick 2014). However, the suggestion has also been made that glycogen and its associated water (1:3 on g per g basis) may represent an important store of available water to combat such dehydration, presumably “released” as glycogen is oxidised during exercise (Maughan et al. 2007; Tam and Noakes 2013). It has been suggested that approximately 1.2 L may become available during a marathon (Maughan et al. 2007).

At rest, water associated with glycogen may be regarded as “specially bound water”. Glycogen when replete constitutes of approximately 2% of muscle wet weight and approximately 5–10% of liver wet weight, but far less after exercise (liver and muscle) and fasting (liver) (Jentjens and Jeukendrup 2003). As such, 6 and 15–30% additional water may exist in muscle and liver respectively (Agius et al. 1991). The fate of such water when liberated within the total body water pool remains unresolved (Cheuvront and Montain 2017; Cotter 2017). This water would likely still contribute to TBW when measured by deuterium oxide (D_2_O) exchange. Therefore, whether or not this water can be an osmotically inactive store is of interest when measuring and determining fluid balance (King et al. 2008). If it is not osmotically active but can be released, then it would be a true store and would add to TBW, potentially offsetting fluid losses during exercise (Maughan et al. 2007). However, if it is in fact osmotically active, then it is simply part of freely available TBW and subject to the normal osmotic equilibria (and not a store per se), which is our hypothesis. If the additional water associated with glycogen is in fact a normal determinant of ICF, it may act purely to support water movement into the ECF in situations of dehydration. The theoretical basis of this has been described by King et al. (2008), although alternative suggestions have been proposed by Maughan et al. (2007).

To test our hypothesis, an investigation was conducted in vitro with glycogen at approximately 2% by mass (i.e., muscle) and approximately 5 and 10% by mass (i.e., liver) together with 150 mmol L^−1^ potassium dihydrogen phosphate (KH_2_PO_4_) (as found intra-cellularly) in a fixed mass of water and osmolality measured to investigate if glycogen provides an osmotically protected space for water. If the glycogen water was not osmotically available, the osmolality of the solution would be greater than expected due to ionic parts from dissociation of KH_2_PO_4_ being unable to access the glycogen bound water. However, if all the water is osmotically active and ionic parts diffuse within the glycogen structure freely, then osmolality would be predicted by normal ionic consideration (hypothesis). Although we could not replicate the true ultra structure of the cell as would be in vivo, there is no reason to believe that the intrinsic physical and chemical behaviour of the component parts (glycogen, potassium ions and phosphate ions) of such a system would be different in vivo from in vitro, but this is a limitation of the testing of our hypothesis. Furthermore, a theoretical exercise was conducted to delineate the changes in ECF and ICF consequent to a situation, where 2 L of sweat loss occurs in scenarios of either significant glycogen-associated water or limited glycogen-associated water.

## Methods

### Experimental design (practical)

This experiment was designed to investigate the effect of electrolyte and varying glycogen concentrations within normal physiological parameters (approximately 2% by mass in muscle and 5 and 10% by mass in liver together with 150 mmol L^−1^of KH_2_PO_4_) to replicate ICF within muscle and liver tissue, on apparent osmolality. Potassium ions and phosphate ions are the major intracellular electrolytes in muscle fibers (King et al. 1978) and liver cells, where glycogen is found. Experiments were conducted in vitro under laboratory conditions. Control solutions of glycogen and KH_2_PO_4_ were tested independently to establish separate osmolalities from which predictions could be established for comparison against measured osmolalities in solutions containing both glycogen and KH_2_PO_4_ at identical to the standard concentrations. We accept that glycogen in vivo would comprise a variety of molecules of differing molecular weight depending on the layering of each unit. This should not interfere with the testing of our hypothesis, because pure glycogen solutions were used as a control. It is possible that there may not be a full response to the theory of dilute solutions for macromolecules in vitro and in vivo. However, this area has been researched and several publications detail theoretical justifications for actual molecular behaviour (Isihara and Guth 1967; Wills et al. 1993). It is relevant that glycogen as a macromolecule has such a large molecular mass in the order of 10^5^–10^6^, depending on actual layering to approach a finite limit. The fact that it is largely neutral, such that in the system tested here at 2 and 10% glycogen with a far greater mass of solvent (water), the solution of glycogen was not likely to be complicated by sufficient perturbations to render data to be confounded in interpretation, or by extension, from in vitro to in vivo. For example, if the granules as visualised by biopsy are considered to have been hydrated spheres in vivo, then the osmotic response as a colligative property is quite minimal (as can be calculated knowing the actual glycogen content of a fibre or cell and also as demonstrated by measurement as shown by Oakley and Young (1936) and indeed the samples as used by us for our study).

#### Preparation of solutions

Two separate samples of glycogen (sample A and B, each from a different phial) from bovine liver were obtained from Sigma-Aldrich (G0885-1g, Type IX, Sigma-Aldrich Company Ltd, Dorset, UK). KH_2_PO_4_ was obtained from Sigma-Aldrich (potassium phosphate monobasic, P5655-100g). Purified water was obtained from Pure Klenz (Pure Klenz, Purified Water conductivity < 1 µs, pureklenz.com, Watford, UK).

Quantities of ingredients used to formulate solutions are shown in Table 1.

**Table 1:** Ingredients of the solutions tested

Condition	Solute (g)		Water (g)
Control solutions			
Control	–		10.00
150 mmol L^−1^KH_2_PO_4_	0.204		10.01
2% by mass glycogen (sample A)	0.201		10.06
5% by mass glycogen (sample A)	0.500		10.04
10% by mass glycogen (sample A)	1.003		10.01
10% by mass glycogen (sample B)	0.498		5.00

	KH_2_PO_4_ (g)	Glycogen (g)	Water (g)
Mixed solutions of simulated muscle and liver			
KH_2_PO_4_ and 2% by mass glycogen (simulated muscle from sample A)	0.205	0.201	10.06
KH_2_PO_4_ and 5% by mass glycogen (simulated liver from sample A)	0.204	0.500	10.04
KH_2_PO_4_ and 10% by mass glycogen (simulated liver from sample A)	0.203	1.003	10.01
KH_2_PO_4_ and 10% by mass glycogen (simulated liver from sample B)	0.103	0.498	5.00

Control solutions of ingredients and mixed solutions of ingredients (simulated muscle and liver) were all prepared using purified water. Solution of ingredients was ensured by rotary fluid currents using a magnetic flea (Scilogex Blue Spin, Connecticut, USA). In combined conditions, glycogen and then KH_2_PO_4_ were added to either 5 or 10 g of purified water to make the solution (Table 1). The pH of the mixed solutions was between 4.2 and 4.6. Solutions were prepared at a room temperature of 22 °C. All reagents and water used were pre-equilibrated to this temperature. No problems were encountered with solution of glycogen or KH_2_PO_4_, and the final solutions were all transparent with no visible particulates. All measurements were made within 60 min of solution.

#### Analysis of solutions

Osmolality was measured by freezing point depression using a calibrated osmometer (Gonotech Osmomat 030-D, 040906, Germany). The osmometer was calibrated against two reference points, using deionised water (0 mOsmol kg^−1^ H_2_O) and a known standard [300 mOsmol kg^−1^ H_2_O (NaCl/H_2_O, Gonotech Calibration Standard, Germany)]. 50 µL of the known standards were pipetted, using an air displacement pipette (Gilson, France) into a micro-container and placed onto the analysis tip of the osmometer, and then placed in the freezing unit. Once calibrated, the standard solution and deionised water were put through as samples, with successful calibration accepted if the values reported were < 2% of the known solutions. The osmometer has a coefficient of variation of 0.9 ± 0.5%, calculated from a known standard solution analysed ten times. 50 µL of prepared samples were analysed in the same manner.

#### Predicted osmolality in simulated muscle and liver solutions

Predicted osmolality in simulated muscle and liver solutions was based on simple summation of the osmolar constituents, exclusively glycogen at approximately 2, 5, and 10% by mass and 150 mmol L^−1^ KH_2_PO_4_. Summation was made on the basis of (a) total water accessibility and (b) limited water accessibility of electrolyte (KH_2_PO_4_) to that mass of water associated with glycogen (assumed as 3:1) (Maughan et al. 2007).

### Experimental design (theoretical)

This was designed to simulate the consequences of exercise induced 2 L sweat loss on changes in TBW, ECF, and ICF in scenarios, where (a) glycogen reserves are full (600 g in total) and (b) where glycogen reserves are low (100 g in total) (Bergström et al. 1967; Detko et al. 2013). An assumed starting status of 42 L TBW distributed as 14 L ECF and 28 L ICF was made (Chawla 2014). The impact of 2 L sweat loss on subsequent movements of water between ECF and ICF was calculated based on osmotic considerations involving (a) considerable glycogen use from full reserves and (b) very limited glycogen use from low glycogen reserves.

#### Calculation of water movements in the theoretical model

Calculations were undertaken on an excel spreadsheet in which a normal TBW (42 L) was assumed to be the composite of two compartments in osmotic equilibrium, ECF (14 L) and ICF (28 L). Initial osmotic equilibrium was set at 285 mOsmol kg^−1^ H_2_O in both compartments and the assumption was made that sodium (Na^+^) was purely 140.0 mmol L^−1^ ECF and K^+^ was 5.0 mmol L^−1^ ECF and 140 mmol L^−1^ ICF at the initial steady state. The scenario of a given sweat loss (2 L) at the fixed concentration of Na^+^ at 40 mmol L^−1^ and K^+^ at 5 mmol L^−1^ was considered typical for modest exercise. The redistribution of water between ICF and ECF consequent to a 2 L sweat loss assumed that the muscle/liver ICF lose glycogen during exercise, such that only glycogen is lost (by oxidation) and that water redistribution occurs as a consequence of sweat loss and osmotic equilibrium. Thus, the loss of sweat initially results in loss of ECF volume, and since sweat [Na^+^] is hypotonic to ECF, [Na^+^] in ECF rises. In the model, no net change in [K^+^] occurs, because sweat and ECF were set to identical concentrations. Consequently (due to loss of hypotonic sweat of low [Na^+^]), there is osmotic imbalance between ICF and a hyperosmolar ECF. Water redistribution then ensues such that there is a net movement of water to ECF from ICF until osmotic equilibrium is achieved (at a slightly higher level than 285 mOsmol kg^−1^ H_2_O). Assumptions involving water associated with glycogen in the special cases discussed later involve the standard basal state as described above as well as a glycogen-depleted state, where TBW and ICF are lower (due to less water associated with a reduced level of intracellular glycogen). Essentially, the rationale for water movements in these two scenarios follow the same osmotic rules following an identical sweat loss.

## Results

### Osmolality of control solutions

The measured osmolalities of control solutions (see Table 1) of KH_2_PO_4_, glycogen, and water are shown in Table 2.

**Table 2:** Osmolality of control glycogen and KH_2_PO_4_ solutions

Solution composition	Measured osmolality (range) (mOsmol kg^−1^ H_2_O)
150 mmol L^−1^KH_2_PO_4_	261.3 ± 3.1 (257–266)
2% by mass glycogen (sample A)	5.8 ± 4.3 (2–11)
5% by mass glycogen (sample A)	4.0 ± 6.9 (0–12)
10% by mass glycogen (sample A)	6.3 ± 5.1 (2–12)
10% by mass glycogen (sample B)	64.5 ± 2.1 (63–66)
Purified water (control)	0 (0–0)

The measured osmolality of 150 mmol L^−1^ KH_2_PO_4_ was found to be 261.3 ± 3.1 mOsmol kg^−1^ H_2_O. The measured osmolality of purified water was found to be 0 mOsmol kg^−1^ H_2_O. The osmolality of solutions of 2, 5, and 10% Glycogen (sample A) was found to be 5.8 ± 4.3, 4.0 ± 6.9, and 6.3 ± 5.1 mOsmol kg^−1^ H_2_O. Solutions of 10% Glycogen (sample B) were found to be 64.5 ± 2.1 (range 63–66) mOsmol kg^−1^ H_2_O.

### Theoretical and measured osmolality of simulated muscle and liver solutions

The predicted (total water accessibility and limited water accessibility of electrolyte) and measured osmolalities of a combination of KH_2_PO_4_, glycogen and water in the “simulated muscle” (~ 2% by mass glycogen A) and in the “simulated liver” (~ 5 and 10% by mass glycogen A and ~ 10% by mass glycogen B) are shown in Table 3.

**Table 3:** Predicted and measured osmolality of simulated muscle and liver solutions

Solution (150 mmol L^−1^ KH_2_PO_4_ plus glycogen mass)	Predicted osmolality (mOsmol kg^−1^ H_2_O)		Measured osmolality (mOsmol kg^−1^ H_2_O)
	Total water accessibility	Limited water accessibility	
Simulated muscle (2% by mass glycogen A)	267	282	265.0 ± 4.7 (260–270)
Simulated liver (5% by mass glycogen A)	265	321	280.0 ± 2.6 (278–283)
Simulated liver (10% by mass glycogen A)	267	407	283.3 ± 4.0 (279–284)
Simulated liver (10% by mass glycogen B)	326	407	348.7 ± 4.7 (345–354)

The osmolality of a combination of KH_2_PO_4_, glycogen A, and water in the “simulated muscle” (~ 2% by mass glycogen) was 265.0 ± 4.7 mOsmol kg^−1^ H_2_O. The osmolality of a combination of KH_2_PO_4_, glycogen A, and water in the “simulated liver” (~ 5% by mass glycogen) was 280.0 ± 2.6 mOsmol kg^−1^ H_2_O. The osmolality of a combination of KH_2_PO_4_, glycogen A, and water in the “simulated liver” (~ 10% by mass glycogen A) was 283.3 ± 4.0 mOsmol kg^−1^ H_2_O. The osmolality of a combination of KH_2_PO_4_, glycogen B, and water in the “simulated liver” (~ 10% by mass glycogen) was 348.7 ± 4.7 mOsmol kg^−1^ H_2_O (Table 3).

## Discussion

The aim of this study was to determine whether or not water associated with glycogen can be an osmotically inactive store, as previously debated by King et al. (2008) and Maughan et al. (2007). Given that this study showed that all water appeared accessible, glycogen water is not a reservoir with the ability to add to TBW when glycogen is oxidised; thus, glycogen water appears osmotically active, simply part of freely available TBW and subject to the normal osmotic equilibria. To this end, we accept our hypothesis. Water associated with glycogen is in fact a normal determinant of ICF, supporting water movement into the ECF in situations of dehydration.

Data from the control solutions of KH_2_PO_4_ and glycogen show the osmolalities expected by calculation. The osmotic coefficient of KH_2_PO_4_ is approximately 0.85 and thus a 150 mmol L^−1^ solution when dissociated would be expected to have an osmolality of approximately 260 mOsmol kg^−1^ H_2_O (i.e., 150 × 2 × 0.85), as verified by testing. Glycogen, a giant molecule, would be expected to have a low osmolality, because glucose units within its structure are bonded α1–6 or α1–4. Indeed, Sample A at 2, 5, and 10% showed only very low osmolalities and sample B at 10% also showed low osmolality. Both values were consistent with the molecular structure of glycogen. It is possible that glycogen sample B, having a slightly higher osmolality than sample A, contained a small amount of free glucose. A solution of pure glucose at 2 and 10% would have expected osmolalities of approximately 110 and 550 mOsmol kg^−1^ H_2_O, respectively (2 g of glucose would yield approximately 11.1 moles glucose, and if dissolved in 10 g water would be equivalent to 111 mOsmol kg^−1^ H_2_O assuming perfect solution, 10 g of glucose would be 5 × greater in osmolality). In early work, Oakley and Young (1936) used collodion membranes to measure the osmotic pressure of glycogen and were using glycogen dissolved in calcium chloride for these measurements. Their experiments were not designed to test for “protected” space but to establish if values obtained for molecular weight were comparable to other chemical methods. Reported mean values were 2 × 10^6^.

The results from the simulated muscle and liver solutions show clearly that measured osmolalities are nearly predictable from the component parts of water, KH_2_PO_4,_ and glycogen and this was true at 2, 5, and 10% simulation for glycogen Sample A and at 10% for Glycogen Sample B (sample with the higher basic osmolality). These data indicate that the relatively small (to glycogen) sized ions of K^+^ and PO4^−^ have good access to all the water in the test sample including that associated with the glycogen. Shown in Table 3 are the predicted osmolalities assuming limited accessibility (i.e., glycogen has protected water) and it can be seen that actual measured osmolalities were all lower than these values. Data for glycogen Sample A at 2% concentration showed no evidence for such protected water. For glycogen Sample A at 5 and 10% and Sample B at 10%, a small amount of protected water was evident. This equates to no more than about 10–20% of glycogen water at 5 and 10% concentrations of glycogen. Taken together, these data suggest that there is no absolute evidence for substantive protected water associated with glycogen in vitro. This is likely also to be the case in vivo given the method of glycogen extraction (e.g., method of extraction was probably aqueous extraction and molecular filtration; thus, no harm comes to glycogen). Glycogen molecules being of very large mass and contributing little to overall osmolality at the concentrations tested would not compromise the measured osmolality of the potassium and phosphate ions unless there was “protected” water.

The findings from this study can likely be extended from in vitro to in vivo. Marchand et al. (2007) and Graham et al. (2010) demonstrated that glycogen found in muscle fibers exists as glycogen granules in their final preparation. The techniques used were based on biopsies of muscle to obtain a sample from which a representation of the ultrastructure of the fibre was obtained by dehydration and subsequent organic staining. This work demonstrated the fact that single giant molecules (of varying layer and size, originating presumably from a glycogenin starter) are the usual presentation of “glycogen” in such prepared samples and by extension (when hydrated) also in the fibre in vivo.

It has been suggested that protected water could provide rehydration when hypohydrated, especially when water loss has occurred through sweat loss (Maughan et al. 2007). Our data would indicate that such water is likely to be of little or marginal significance, if any, and that normal osmotic constraints are observed over physiological concentrations of glycogen. It would be worthwhile to consider approaches in vivo where accurate and precise osmotic pressure changes simultaneous with changes in cell glycogen and water were made, but this would be difficult in humans.

It is possible, however, to consider the theoretical implications of water associated with glycogen and the normal distribution of water between ECF and ICF in given exercise situations. In this case, consider the redistribution of water (from ICF to ECF) as a consequence of typical sweat loss as would occur during modest physical activity. In addition, the specific fate of glycogen-associated water can be considered (liver and muscle only). These considerations can be modelled against typical but different glycogen contents (in muscle or liver).

**Fig. 1:**
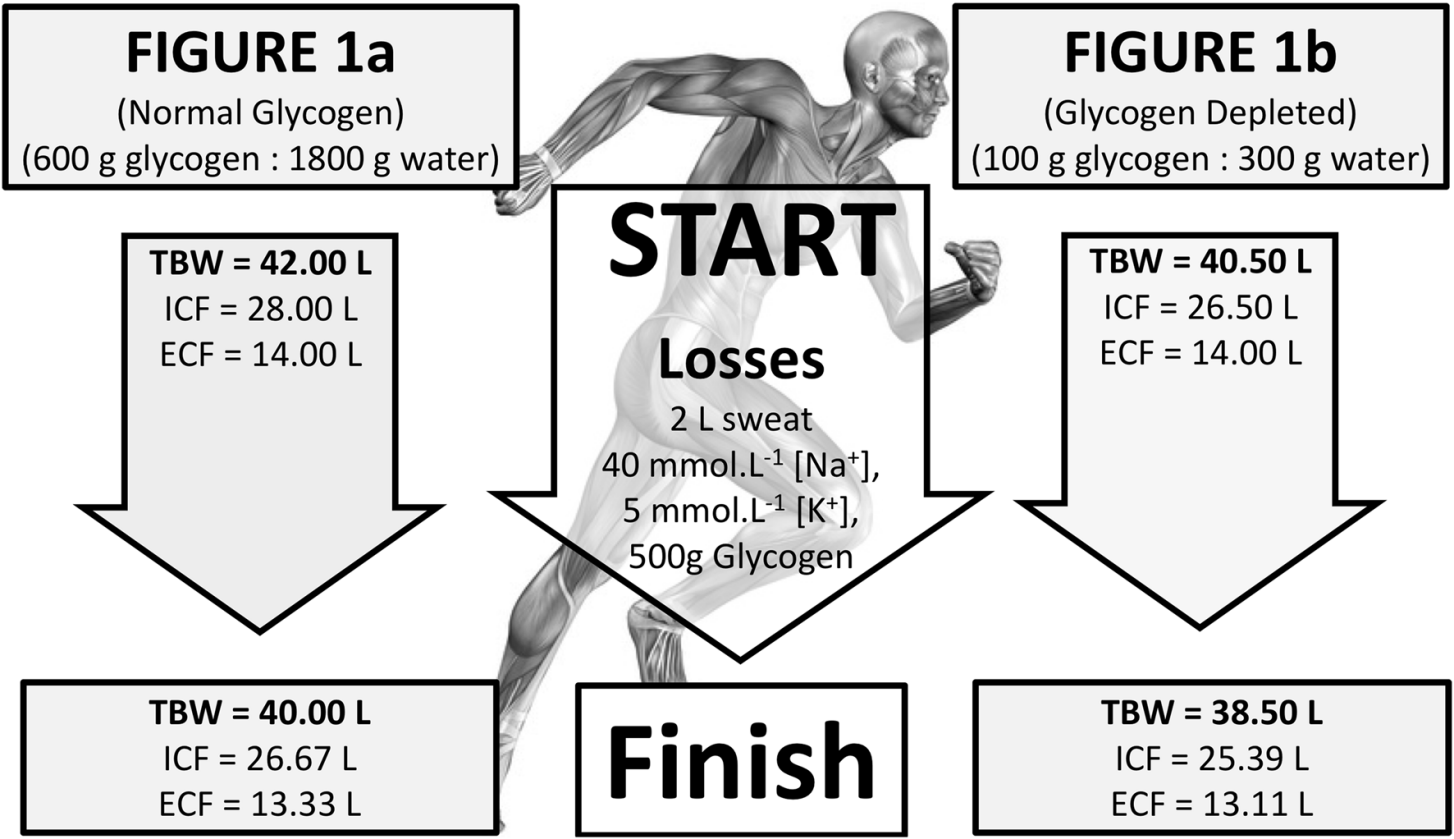
Theoretical change in TBW, ICF, and ECF water stores during exercise for **a** normal glycogen stores prior to exercise and **b** depleted glycogen stores prior to exercise

Two opposite situations relative to glycogen storage, one where glycogen stores are full (i.e., 600 g glycogen:1800 g glycogen-associated water), the other where glycogen stores are significantly depleted (i.e., 100 g glycogen:300 g glycogen-associated water) are depicted in Fig. 1. In both scenarios, it is assumed that osmotic stability prevails, prior to the onset of exercise in which significant use of glycogen may occur. In the case of full glycogen stores (Fig. 1a), the initial ICF (42 L) is probably 1.5 L larger than would be for glycogen depletion (Fig. 1b; ICF, 40.5 L), where only 0.3 L of osmotically active water space could be assigned. In both situations, the majority of this osmotically active, glycogen-associated water would be within liver and muscle. This water still forms part of the osmotic equilibrium between ICF and ECF, so when hypotonic sweat loss occurs during exercise, there would be the normal redistribution of water between compartments, thus maintaining equilibrium. What is apparent by calculation is that the loss of ICF to maintain equilibrium is different for the two glycogen starting points. However, the difference is trivial.

The impact of having more glycogen-associated water when starting exercise with normal glycogen confers an advantage of only an additional 0.22 L on ECF compared to a similar sweat loss of 2.0 L when starting exercise glycogen deplete. Since plasma (approximately 3.5 L) is usually in osmotic equilibrium with other extracellular compartments, this represents about a 1.57% advantage (e.g., approximately 55 mL extra plasma volume) if indeed, a greater plasma volume is preferable.

The theoretical aspects discussed above assume that the muscle/liver ICF lose glycogen during exercise, such that only glycogen is lost (by oxidation) and that water redistribution occurs as a consequence of sweat loss and osmotic equilibrium. Thus, the ICF of either liver or muscle would enter a state in which some of the water and K^+^ recently associated with glycogen is then without its glycogen, but the cells are in osmotic equilibrium with ECF. It is well established that cells swell when glycogen repletion occurs and shrink when depletion occurs to specific steady-state volumes (Agius et al. 1991). Thus, the reestablishment of glycogen-dependent cell volume either occurs concurrently with the above osmotic considerations, is delayed until exercise ceases when sweat loss may be minimal or is affected by carbohydrate feeding in which the potential for glycogen repletion is made. If it is accepted that following glycogen loss, both redistribution of water and resetting of cell volume occur, then the following could happen. Not only would the simple osmotic movements outlined above cause compartment change in ECF and ICF, but in addition further loss of water and electrolytes (K^+^ and counter anion) previously associated with that glycogen would also be redistributed and this would need osmotic balance to be maintained. This would be directly related to the mass of glycogen metabolised during exercise. For example, if overall 500 g glycogen was to be used and cell volume normalised to the resulting residual glycogen content, then some 1.5 L of water and its associated ICF electrolyte content (about 140 mmol L^−1^ K^+^ and 140 mmol L^−1^ anion, say 210 mmol K^+^ and 210 mmol anion in total) would need to be redistributed. As a consequence, hypotonic sweat loss and the simple osmotic redistribution of water (from ICF) would result in an ECF (inclusive of plasma) change in K^+^ mmol L^−1^ from 5.0 to 4.6 mmol L^−1^; thus, transfer of only 5 or 6 mmoles from ICF would be needed to restore K^+^. If 210 mmoles K^+^ were to be distributed to all the ICF (likely since ICF [K^+^] > ECF [K^+^], and ECF [K^+^] is tightly regulated), the resulting internal change would be from 140 to 148 mmol L^−1^. Urine losses could perhaps account for only 20 mmol L^−1^ and thus of little consequence. As such, the maximum benefit if this 1.5 L of glycogen water were to be distributed purely to osmotic balance may only be 54–64 mL to ECF (approximately 0.40–0.45%) and the majority of fluid to ICF. Clearly then, glycogen water is unlikely to be of significant hydration benefit to ECF and plasma water (vascular volume), as evidenced on either practical (glycogen water is osmotic) or theoretical grounds (redistribution based on osmotic considerations of K^+^).

In conclusion, glycogen water does not appear to be a reservoir per se during dehydration, given that the greater part of water appeared accessible at varying concentrations. The majority of water associated with glycogen appears osmotically active, especially so at lower glycogen concentrations, and will have little influence preserving TBW during exercise induced dehydration [e.g., replacing sweat (fluid) loss], which should be considered when monitoring fluid balance in situations of dehydration.

## Abbreviations

ECF: Extracellular fluid
ICF: Intracellular fluid
KH_2_PO_4_: Potassium dihydrogen phosphate
K^+^: Potassium
Na^+^: Sodium
TBW: Total body water
